# Chemokine polyreactivity of IL7Rα^+^CSF-1R^+^ lympho-myeloid progenitors in the developing fetal liver

**DOI:** 10.1038/srep12817

**Published:** 2015-08-03

**Authors:** Katja Kajikhina, Fritz Melchers, Motokazu Tsuneto

**Affiliations:** 1 Max Planck Institute for Infection Biology, Chariteplatz 1, 10117 Berlin, Germany

## Abstract

In murine ontogeny, fetal liver is the major hemato- and B-lymphopoietic site until birth. Hematopoiesis develops in largely non-hematopoietic niches, which provide contacts, chemokines and cytokines that induce migration, residence, proliferation and differentiation of progenitors. Within early multipotent progenitors an IL7Rα^+^CSF-1R^+^ subset expressed a mixture of lymphoid- and myeloid-specific genes and differentiated to lymphoid and myeloid lineages *in vitro*. By contrast, IL7Rα^+^ cells were lymphoid-committed, and CSF-1R^+^ cells were erythro-myeloid-restricted. To respond to a multitude of chemokines single biphenotypic cells expressed CXCR4 and as many as five other chemokine receptors. The monopotent IL7Rα^+^ and CSF-1R^+^progenitors all expressed CXCR4, and mutually exclusive, more restricted sets of the analysed five chemokine receptors. This study proposes that chemokine polyreactive, cytokine-bipotent and monopotent progenitors transmigrate through LYVE-1^high^ endothelium, attracted by selected chemokines, and reach the IL7- and CSF-1-producing ALCAM^high^ mesenchymal niche, attracted by other sets of chemokines, to differentiate to B-lymphoid respectively myeloid cells.

During ontogeny of the mouse several sites contribute to hematopoiesis^1^. The first wave of hematopoiesis is initiated at the extra-embryonic yolk sac (YS) around embryonic day (E)7, where ξ-globin^+^ enucleated erythrocytes are produced. The second wave starts at the yolk sac with definitive erythro-myeloid progenitor (EMP) cells^2^,^3^ giving rise to red blood cells, specific adult-type myeloid and lymphoid cells, such as megakaryocytes, platelets and special subtypes of long-lived myeloid subsets such as liver Kupffer cells, brain microglia and epidermal Langerhans cells^4^,^5^,^6^,^7^,^8^. The YS generates furthermore immune-restricted, lymphoid primed progenitors which establish a transient wave of lymphoid and of c-myb-independent resident myeloid cells prior to emergence of pluripotent hematopoietic stem cells^7^,^9^,^10^,^11^. The EMP-derived erythroid cells are characterized by specific gene expression such as ßH1-globin^12^ and colonize the fetal liver around E10.5 where final lineage differentiation takes place^13^.

At E8.5 a third, now intra-embryonic, definitive hematopoiesis is established in the aorta, gonad and mesonephros (AGM) region and umbilical and vitelline arteries^14^,^15^. Blood circulation is established, and first pluripotent hematopoietic stem cells (pHSCs) derived from the hemogenic endothelium are found at E10.5^16^,^17^,^18^. Clusters of hematopoietic stem and progenitor cells accumulate in the arterial lumen prior to the onset of circulation and colonization of the fetal liver^19^.

Definitive AGM-derived hematopoiesis has been studied extensively. Long-term reconstituting hematopoietic stem cells (LT-pHSCs), characterized as LSK CD150^+^CD48^−^20, are capable of life-time repopulation of lethally irradiated recipient mice and can generate different lineages of specialized blood cells – red blood cells, platelets, myeloid and lymphoid cells- with the possible exception of long-lived myeloid cells. As hematopoietic development proceeds, pHSCs lose their long-term reconstitution capacity, while retaining multipotency and homing capacity to bone marrow, still able to short-term repopulate irradiated blood system. Next, LSK CD150^−^CD48^+^ Flk2^+^ multipotent hematopoietic progenitors (MPP) with the capacity to generate myeloid and lymphoid cell lineages develop^21^.

Specific transcription factor expression marks distinct stages of lymphoid and myeloid lineage differentiation^22^. EBF expression and high levels of Pax5 commit IL7Rα^+^ CLP to B-lymphoid lineage differentiation^23^, while low levels of Pax5 stabilize an earlier, biphenotypic myeloid/B-lymphoid stage of development^24^. PU.1 coordinates myeloid differentiation^25^, while E2A is an essential factor for lymphoid-primed MPP expansion^26^. GATA-1 is required for normal erythropoiesis^27^. Later lineage restricted stages express transcription factors such as Hes1, which is a downstream target of Notch signaling. It was shown to inhibit erythroid/megakaryocytic differentiation by suppression of GATA-1^28^ and to promote T cell specification and commitment, negatively regulating PU.1 and C-EBP/ α activity^29^,^30^.

Early progenitors at points of lympho-myeloid lineage decisions have previously been described in fetal and adult hematopoiesis^11^,^31^. Cell fate choice of uncommitted progenitors has traditionally been explained by hematopoietic cell-intrinsic, stochastic models^32^. Later studies provided evidence, that extrinsic factors, e.g. cytokines produced by microenvironments of hematopoiesis, influence lineage specific differentiation^33^. Two cytokines are known to be essential for lymphoid and myeloid lineage differentiation in the mouse. IL7 is the major non-redundant cytokine driving lymphoid differentiation by triggering intracellular signaling and changes in gene expression programs through the IL7Rα to all stages of early B, T and NK cell differentiation^33^,^34^. CSF-1 stimulates myeloid differentiation to tissue macrophages and osteoclasts through signaling via the CSF-1R^35^,^36^,^37^,^38^,^39^,^40^.

Here, we provide evidence that an early progenitor subset expresses both IL7Rα and CSF-1R on the same cell, being capable to differentiate both to lymphoid and myeloid cells. Thus, the question arises, how these cytokine-bipotent IL7Rα^+^-CSF-1R^+^ progenitors are instructed to become committed to one of the two lineages by their non-hematopoietic and hematopoietic microenvironments.

In E14.5 fetal liver the two cytokines, IL7 and CSF-1 have previously been found to be produced by non-hematopoietic, VCAM^+^CD105^low^ALCAM^high^ mesenchymal stromal cells, but not by VCAM^+^CD105^high^Lyve1^high^ endothelial cells^41^. Here, we extend this analysis of non-hematopoietic stromal cells for their function in support of homing and development of early progenitors on the branching point of the lympho-myeloid lineage decision in fetal liver at E13.5 and E16.5.

## Results

### Development of hematopoietic stem cells and progenitors in the fetal liver

**Figure 1:**
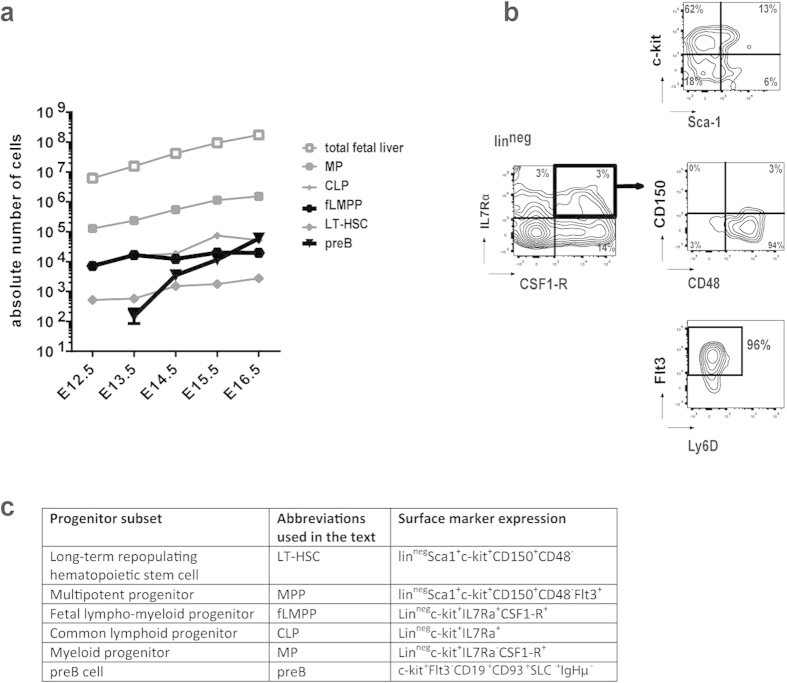
Quantitation and characterization of early hematopoietic progenitor subsets within the developing fetal liver of the murine embryo between E13.5 and E16.5. (**a**) Developmental kinetics of hematopoietic stem and progenitor cells and B cell precursors within the developing fetal liver between E12.5 and E16.5. Phenotypes of subsets were characterized as following: CSF-1R^+^: lin^neg^c-kit^+^IL7R-CSF-1R+; IL7Rα^+^: lin^neg^c-kit^+^IL7Rα^+^CSF-1R^−^; CSF1-R^+^IL7Rα^+^: lin^neg^kit^+^IL7Rα^+^CSF-1R^+^; pHSC: LSK CD150^+^CD48^−^; preB: CD19^+^IL7R^+^SLC^+^IgM^−^; each data point shows mean±SD from 6–10 embryos. (**b**) Phenotypic characterization of hematopoietic fractions within murine fetal liver at E13.5 by FACS describes a biphenotypic lin^neg^IL7Rα^+^CSF-1R^+^ progenitor subset that expresses c-kit, Sca1, CD45, Flt3, CD48, no CD150, no Ly6D. (**c**) Summary of the phenotypes that were analysed in the present study and the abbreviation for each subset used in all parts of the manuscript.

We analysed by flow cytometry the developing numbers of early hematopoietic stem and progenitor cell subsets within the growing fetal liver between E12.5 to E16.5: LT-pHSCs (LSK CD150^+^CD48^−^), MPPs (LSK CD150^−^CD48^+^Flt3^+^), myeloid progenitors (lin^neg^c-kit^+^Flt3^+^CSF-1R^+^IL7Rα^−^) and CLP-like progenitors (lin^neg^c-kit^+^Flt3^+^CSF-1R^−^IL7Rα^+^) (Fig. 1a).

One fetal liver at E13.5 contained less than 10^3^ LT-HSCs, and this number remained so low until E16.5. Numbers of MPPs and CLPs were constantly 10-fold, and those of myeloid progenitors (MPs) 100-fold higher. All three subsets increased only slightly until E16.5, although the total number of cells in the organ doubled every day (Fig. 1a). By contrast, the number of c-kit^+^Flt3^−^CD19^+^CD93^+^SLC^+^IgHμ- preBI cells (phenotype as shown in Supp. Fig. 1b) increased exponentially, by 400-fold between E13.5 and E16.5 (Fig. 1a). This confirmed our earlier findings, in which the kinetics of B-cell precursor development including the stepwise V(D)J-rearrangements at the IgH-locus have been quantitated^41^.

### An early subset displays early progenitor markers and expresses both IL7Rα and CSF-1R

A progenitor subset between E12.5 and E16.5 was found to express both IL7Rα and CSF-1R (Fig. 1b). The majority of these cells was negative for lineage markers (CD3, CD19, Gr1, Ter119, F4/80), were heterogeneous in their Sca1 expression, and almost all of them (~96%) expressed c-kit, CD48, Flt3, no CD150 and no Ly6-D (an early B-cell specification marker^42^) (Fig. 1b). This subset was exclusively detected within the classic multipotent progenitor (MPP) subset^43^, no overlapping was found with the subsets of LT-HSCs and other hematopoietic progenitor subsets as shown in Supp. Fig. 2. At E13.5 the frequency of these biphenotypic progenitors was approximately 10^4^ in 10^7^ fetal liver cells. By E16.5 the total number of fetal liver cells had increased a 1000-fold, while the number of double cytokine receptor-expressing cells remained unchanged (Fig. 1a). The analysed phenotypes and the corresponding nomenclature are summarized in Fig. 1c.

We conclude that these biphenotypic fLMPP cells are phenotypically close to the classic LSK CD150^−^CD48^+^Flt3^+^ MPP fraction (Supp. Fig. 2) not yet expressing any lineage commitment markers and can be expected to be reactive to the myeloid cytokine CSF-1, as well as to the lymphoid IL7.

### IL7Rα^+^CSF-1R^+^ progenitors express lymphoid and myeloid transcription factors

**Figure 2:**
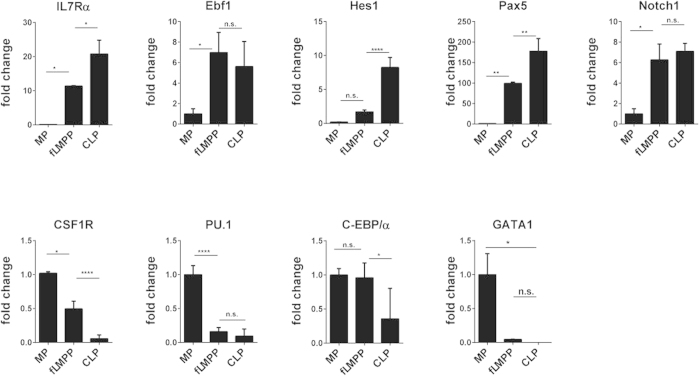
Lineage-specific transcription factor expression in IL7Rα^+^ and CSF1-R^+^ progenitors. Real-time PCR based transcription factor expression analysis of sorted CLP, MP and fLMPP subsets from murine fetal liver at E13.5. Reactions were performed with template cDNA transcribed from total 300 ng RNA each. Results show mean ± SD, calculated relative to GAPDH expression using the ΔΔCt method. Expression levels are shown as fold change compared to the MP fraction. One-way ANOVA test with multiple comparisons was performed to test the statistical significance (95% CI).

We aimed to test the lympho-myeloid character of the fLMPP subset and analysed the expression of lineage-specific transcription factors in the sorted fLMPP progenitors and compared it with the expression levels in CLP or MP cells (Fig. 2). The fLMPP subset was found to express intermediate levels of lineage-instructive transcription factors Ebf1 (B-lineage), C-EBP/α (myeloid), Pax5 (B-lymphoid), and Notch1 (T-lymphoid). Hense, PU.1 (myeloid) and GATA-1 (erythroid) were only expressed in the sorted MP cells. The transcription factor Hes1 acts downstream of Notch driving cells to lymphoid restriction^44^. We found Hes1 to be expressed in considerable levels in the CLP fraction, but at significantly lower levels or not at all in the fLMPP and MP fractions only (Fig. 2). These results suggest that the biphenotypic progenitors express a transcription factor program with the potency of lymphoid as well as of myeloid differentiation.

### IL7Rα^+^CSF-1R^+^ progenitors differentiate to lymphoid and myeloid cells *in vitro*

**Figure 3:**
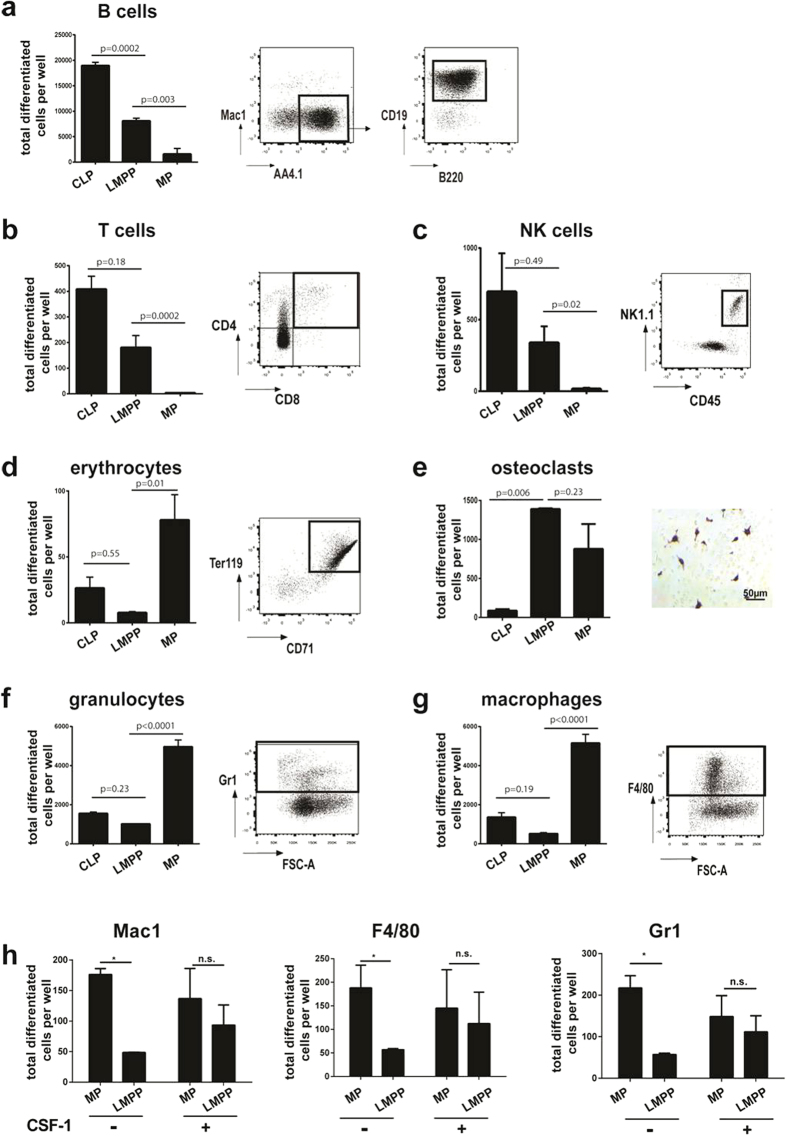
*In vitro* differentiation capacities of sorted progenitors isolated from murine fetal liver at E13.5. (**a**) B-lymphoid differentiation on OP9 stroma cells, supplemented with IL7, (**b**) T cell differentiation on OP9-Δ/Flt3L/IL7, (**c**) NK cell differentiation on OP9/IL4/IL15, (**d**) erythroid differentiation on OP9/SCF/Epo, (**e**) osteoclast differentiation on ST2 stroma, with dexamethasone and vitamine D, numbers of differentiated cells were quantified by TRAP staining, (**f**) and (**g**) myeloid differentiation on CSF-1 deficient OP9 with G-CSF and GM-CSF. Data are shown as mean±SD, representative of three experiments, each performed in triplicates. One-way ANOVA test with multiple comparisons was performed to test the statistical significance (95% CI). (**h**) CSF-1 complementation experiment was performed with 100 sorted cells per well on CSF-1 deficient OP9, media was supplemented with SCF (−) or SCF and CSF-1 (+). A two-tailed unpaired t-test with Holm-Sidak correction for multiple comparisons was used to determine the statistical significance (95% CI).

Next, we tested the capacities of double- and single-cytokine receptor-expressing progenitors to differentiate in culture to lymphoid, myeloid and erythroid lineages. The fLMPP cells developed to CD19^+^B220^+^ B-cells, CD4^+^CD8^+^ T-cells and to NK1.1^+^ NK-lymphoid cells (Fig. 3a–c). In addition, they also gave rise to TRAP^+^ osteoclasts under appropriate culture conditions (Fig. 3e). On the other hand, sorted MP cells developed to myeloid cells in the presence of CSF-1, but did not differentiate to lymphoid lineages. Vice versa, in the presence of IL7, single CLPs differentiated to lymphoid cells, but failed to produce myeloid cells on ST2 stroma with CSF-1.

We have tried, but failed, to expand single fLMPPs by proliferation, followed by differentiation of the proliferated cells either to myeloid or to lymphoid lineage cells. In addition, myeloid or lymphoid colony assays in semisolid media showed plating efficiencies of less than 20%. Therefore even these experiments did not allow us to decide between bi- and monopotency of single fLMPPs.

While our results suggest that the population of double cytokine receptor positive fLMPPs could be bipotent for myeloid and lymphoid differentiation, they do not exclude the possibility that the potency to differentiate to either myeloid or lymphoid lineage cells is a property of separate cells within this population.

We also performed myeloid differentiation cultures on CSF-1-deficient OP9 which produce the myeloid factors G-CSF and GM-CSF and observed that the IL7Rα^+^CSF-1R^+^ progenitors showed only a very low capacity to differentiate to Gr-1^+^ granulocytes and F4/80^+^ macrophages (Fig. 3f,g). Furthermore we analysed the dependency of the myeloid differentiation of fLMPPs on presence of CSF-1 and performed differentiation cultures on CSF1-deficient OP9 supplemented with CSF-1. Figure 3h shows that addition of recombinant CSF-1 could adjust the differentiation capacity of the fLMPP subset to that of the sorted MP progenitors for Gr1^+^, F4/80^+^ and Mac-1^+^ cells.

This indicates that the CSF-1R signaling and presence of CSF-1 is indispensable to induce myeloid lineage differentiation of the analysed lympho-myeloid progenitors. By contrast, MP cells were able to produce high numbers of Gr1^+^ granulocytes and F4/80^+^ respectively Mac1^+^ macrophages also in the absence of CSF-1.

The fLMPP subset also failed to differentiate to Ter119^+^CD71^+^ erythroid cells under appropriate culture conditions (Fig. 3d). Only the MP subset was able to give rise to high numbers of erythroid cells.

We propose therefore that the progenitors expressing both cytokine receptors only possess lymphoid and CSF-1 dependent myeloid capacity lacking the erythroid potential, whereas the MP cells still are capable of giving rise to both myeloid and erythroid lineages, which is also reflected in the higher expression of transcription factors PU.1 and GATA-1 in this fraction. The CLP fraction is lymphoid-committed being capable to efficiently produce B, T and NK cells, but no further blood cell lineages.

### Lymphoid and myeloid progenitors express distinct sets of chemokine receptors, while the lympho-myeloid progenitors express both sets

Progenitors are guided to the supporting microenvironments by chemotaxis. Therefore, double and single cytokine receptor-expressing progenitors from fetal liver between E13.5 and 16.5 were sorted and analysed regarding their expression of known chemokine receptors.

**Figure 4:**
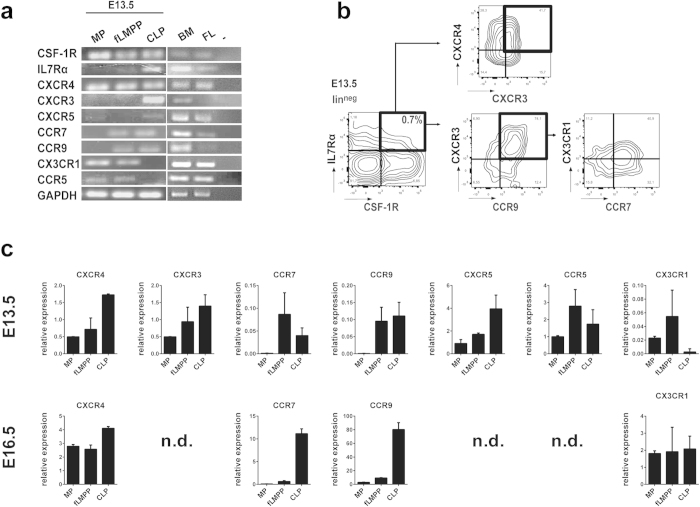
Chemokine receptor expression of biphenotypic IL7Rα^+^CSF1-R^+^ progenitors. (**a**) Expression of chemokine receptors in sorted progenitor fractions. RT-PCR was performed on cDNA template transcribed from 300 ng RNA isolated from fetal livers at E13.5 and E16.5. Sorted fractions: lin^neg^c-kit^+^IL7Rα^+^CSF1-R^−^, lin^neg^c-kit^+^IL7Rα^+^CSF1-R^+^, lin^neg^c-kit^+^IL7Rα^−^CSF-1R^+^; (**b**) FACS analysis of the chemokine receptor expression at E13.5 shows simultaneous surface expression of CXCR4, CXCR3, CCR7, CCR9 and CCR5 on the surface of single IL7Rα^+^CSF1-R^+^ progenitors. (**c**) Real-time PCR based relative quantitation of gene expression levels of the analysed progenitors at E13.5 and E16.5. n.d.: not detected.

At E13.5 a broad range of chemokine receptors was found to be transcribed in fLMPP progenitors, while a more restricted set was detected in single cytokine receptor-expressing cells (Fig. 4a). Thus, CXCR3, CXCR4, CCR2, CCR5, CCR7, CCR9 and CX3CR1 were expressed in IL7Rα^+^CSF-1R^+^ cells at E13.5. By contrast, only CXCR4, CCR5, and CX3CR1 were expressed in CSF-1R^+^ cells, while single IL7Rα^+^ cells expressed CXCR4, CXCR3, CCR2, CCR7 and CCR9. Hence, only CXCR4 was uniformly expressed in all sorted progenitor subsets, while two non-overlapping chemokine receptor sets were selectively expressed in lymphoid- respectively in myeloid-restricted progenitors. We quantitated the levels of the candidate chemokine receptors and found that the fLMPP fraction at E13.5 expressed intermediate levels of the analysed chemokine receptors CXCR4, CXCR3, CCR9 and CXCR5 (Fig. 3c). This fraction furthermore contained the highest amount of transcripts for CCR7, CX3CR1 and CCR5 among the analysed fractions. At E16.5 we found that CXCR4 was still expressed ubiquitously, whereas futher receptor such CCR7 and CCR9 became restricted to the CLP fraction. We could not detect considerable levels of the receptors CXCR3, CCR5 and CXCR5 at E16.5 in any of the fractions.

The surface expression of these candidate chemokine receptors was confirmed by FACS analysis. We observed the lympho-myeloid cells to express the highest levels of chemokine receptors on their surface on E13.5 compared to the lymphoid and myeloid fractions (Fig. 4b). We were able to detect the receptors CXCR4, CXCR3, CCR7 and CCR9 being simultaneously expressed on the lympho-myeloid progenitors (Fig. 4b). This indicates that the analysed chemokine receptors are expressed simultaneously on the surface of single progenitors enabling a single cell to react to several chemokines.

### CXCL12 is the most potent chemoattractant for all three progenitor subsets

We performed *in vitro* migration assays to evaluate the chemotactic response of sorted cytokine-bipotent and –monopotent progenitors to the chemokines which are known to interact with the chemokine receptors described above (Fig. 4).

**Figure 5:**
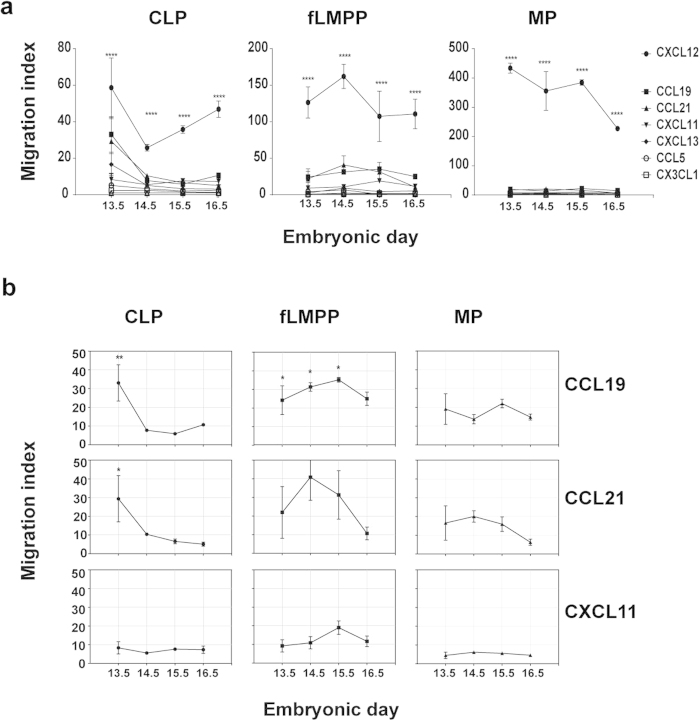
Chemotactic responsiveness of progenitors from E13.5-E16.5 in an *in vitro* transwell assay. Total 10^5^ lin^neg^ cells from fetal livers between E13.5 and E16.5 were inserted into a transwell chamber to assay the *ex vivo* migration capacity of early progenitors to recombinant chemokines (100 ng/ml). The number of migrated cells was normalized to the number of migrated cells in absence of any chemokine in the same experiment (migration index). A two-way ANOVA test with multiple comparisons was performed to test the statistical significance (CI 95%), ****p < 0.0001, **p < 0.01, *p < 0.05.

CXCL12 attracted the highest number of all three progenitor subsets inducing a highly significant response compared to the migration in absence of any chemokine at all times of fetal liver development tested in our experiments, both CLP and MP as well as for the lympho-myeloid fLMPP cells (Fig. 5a).

Of all other tested chemokines only CXCL11 (corresponding with CXCR3), CCL19 and CCL21 (ligands for CCR7) induced specific, though lower chemoattraction of cytokine-bipotent progenitors at E13.5 and E14.5 (migration indices above 10, Fig. 5b). The chemokines CCL19 and CCL21 were chemoattractive to CLPs, but also for the intermediate fLMPPs, consistent with the shown expression of CCR7 in these subsets (Fig. 4). No significant chemoattraction was induced by these chemokines in MP cells. The chemokines CXCL13, CCL5 and CX3CL1 did not induce any significant response in the performed *in vitro* assay in any of the subsets (Supp. Fig. 4).

These data show that the migration capacity *in vitro* to a chemokine in solution does not directly correlate with the expression of a corresponding chemokine receptor on the surface of a cell. Still, the biphenotypic fLMPP progenitors appear to be polyreactive to the chemokines CXCL12 and CCL19/21 and CXCL11, especially at early stages of fetal liver development.

### Endothelial and mesenchymal cells in fetal liver provide distinct sets of chemokines and cytokines

**Figure 6:**
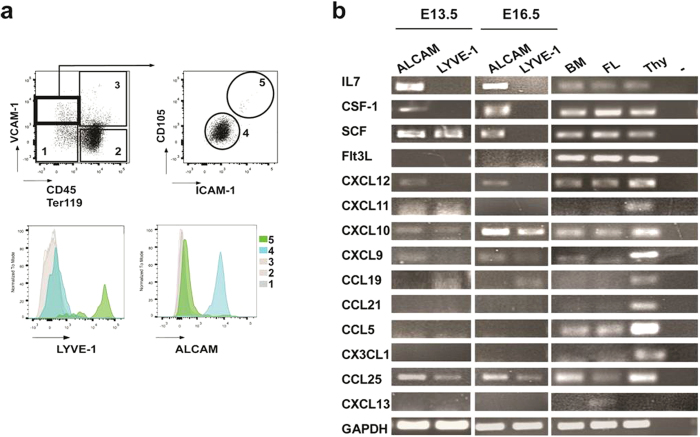
The endothelial and mesenchymal stroma in the developing fetal liver has distinct supporting capacities for hematopoietic development of lymphoid and myeloid progenitors. (**a**) Hematopoietic and stromal compartments within the fetal liver at E13.5. Surface marker analysis by FACS shows two distinct VCAM-1^+^CD45^−^Ter119^−^ populations: CD105^low^ICAM^+^ALCAM^high^ and CD105^high^ICAM^+^Lyve-1^high^, (**b**) Endothelial and mesenchymal subsets were sorted and analysed regarding their chemokine and cytokine expression. Each reaction was carried out on cDNA transcribed from 150 ng RNA from sorted VCAM-1^+^CD45^−^Ter119^−^CD105^low^ICAM^+^ALCAM^high^ or VCAM-1^+^CD45^−^Ter119^−^LYVE-1^high^ fractions isolated from fetal livers at E13.5 and E16.5 and compared to the expression in total adult BM, total adult thymus and total fetal liver E17.5.

In a search for supporting cells, which produce chemokines and cytokines to induce homing and lineage-differentiation, stromal cells have previously been FACS-enriched from E14.5 fetal liver for non-hematopoietic VCAM^+^CD105^high^ endothelial cells and VCAM^+^CD105^low^ liver mesenchymal cells. LYVE-1 was found highly expressed on endothelial, but not on mesenchymal cells, while ALCAM was highly expressed on mesenchymal cells^41^. In this study, we analysed LYVE-1^high^ and ALCAM^high^ cells from fetal liver between E13.5 and 16.5 (Fig. 6).

By RT-PCR analyses we probed these two types of cells for their capacities to express extrinsic factors, which were expected to contribute to the control of early hematopoietic development (Fig. 6b).

As seen previously at E14.5 the myeloid cytokine CSF-1 and the lymphoid IL7 were both produced in ALCAM^high^, but not in LYVE-1^high^ cells from E13.5 to E16.5. By contrast, SCF, which is essential for proliferation of progenitors, was produced by both types of cells at E13.5.

In the present study we found again, that CXCL12 was expressed in ALCAM^high^ mesenchymal, but not in LYVE-1^high^ endothelial cells between E13.5 and E16.5. Hence, any possible endothelial transmigration of progenitors cannot be effected by the direct secretion of CXCL12 by endothelial cells. By contrast, LYVE-1^high^ endothelial cells produced CXCL10, CCL25 and CCL19 between E13.5 and E16.5, while expression of CXCL11 was detected at E13.5, but no longer at E16.5. Hence, at least CXCL10, CXCL11 and CCL19 might be involved in a possible transendothelial migration of cytokine-bi- and –monopotent progenitors into the developing fetal liver between E13.5 and E16.5 as we observed the corresponding chemokine receptors to be expressed and these chemokines induced *in vitro* chemotactic responses in these progenitors.

At E13.5 ALCAM^high^ liver mesenchymal cells produced not only CXCL12, but also CXCL9, CXCL10, CXCL11 and CCL25. CXCL9 and CXCL10 remained expressed at E16.5. CX3CL1 and CCL5 were not found expressed at any time between E13.5 and E16.5. Therefore, at E13.5, bi- and monopotent progenitors could be attracted to IL7 and CSF-1 producing ALCAM^high^ cells by the combination of CXCL12 and these other chemokines.

## Discussion

In our studies we isolated and analysed progenitors, which express either IL7Rα or CSF-1R, or both cytokine receptors together. The biphenotypic progenitors were shown to express CD45, CD48, c-kit and Flt3, but do not express CD150 or any lineage-specific markers. Hence, this marker expression is comparable with the characteristics of the classic MPP compartment^43^. In fact, our analyses indicate that at least 20% of cells within the MPP pool expresses both cytokine receptors together (Supp. Fig. 2). Since the cytokine receptors are expressed at low levels, it cannot be excluded that an even larger percentage of MPPs might express the cytokine receptors at levels not detectable in our FACS experiments.

The biphenotypic progenitors were found to express a collection of lineage specific lymphoid (Ebf1, Pax5, Notch1) and myeloid (C-EBP/α) genes. Our analyses agree with single cell-expression analyses done previously^11^. Our IL7Rα^+^CSF1^−^R^+^ cells were able to differentiate *in vitro* to B, T in response to IL7, and also to NK cells. In response to CSF-1, the same cell population was able to generate macrophages and osteoclasts. Thus, these progenitors were, in fact, cytokine-bipotent, capable of lymphoid and myeloid differentiation upon IL7 and CSF-1 stimulation. Since we were unable to determine this bipotency for differentiation for myeloid and lymphoid cells on a single fLMPP level it should be emphasized that our experiment do not rule out that fLMPPs either in part or totally mixtures of cells with monopotent capacities.

Interestingly, CSF-1R^+^ progenitors differentiated *in vitro* to Mac-1^+^, Gr-1^+^ and F4/80^+^ myeloid cells also in the absence of CSF-1 (on CSF-1 deficient OP9 stromal cells with G-CSF and GM-CSF) and they also produced Ter119^+^CD71^+^ erythrocytes under erythroid culture conditions. By contrast, no myeloid cells differentiated from the IL7Rα^+^CSF1-R^+^ progenitors in the absence of CSF-1. This dependency on CSF-1 was also proved by *in vitro* cultures on CSF-1 deficient OP9 cells that were complemented with recombinant CSF-1, where similar numbers of Gr1+, F4/80 and Mac1+ cells were produced from sorted fLMPPs compared to the cultures starting with sorted MP cells (Fig. 3h).

**Figure 7:**
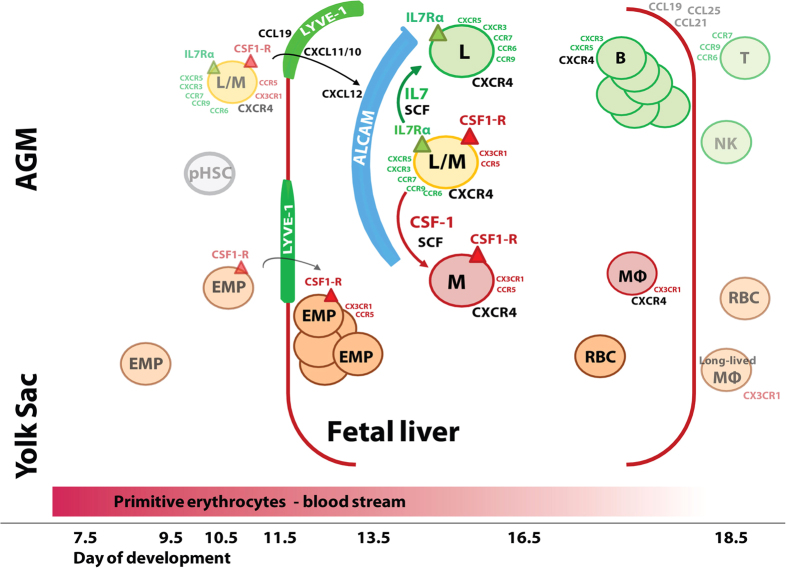
Proposed model of lympho-myeloid lineage differentiation in the developing fetal liver of the mouse. Two hematopoietic lineages populate the murine fetal liver at E13.5. The majority (10^5^ cells) are yolk sac-derived, CXCR4^+^CCR5^+^CX3CR1^+^CSF-1R^+^ EMPs, characterized by high expression of PU.1 and GATA-1 and by their capacity to develop *in vitro* to erythrocytes and myeloid cells even in the absence of CSF-1. The second lineage is AGM pHSC-derived and contains 10-fold lower numbers of cytokine-bipotent IL7Rα^+^CSF-1R^+^cells. These lympho-myeloid cells express lymphoid chemokine receptors CXCR3, CXCR4, CXCR5, CCR6, CCR7 and CCR9, as well as myeloid-directed CCR5 and CX3CR1 on the same surface. This progenitor pool also contains cytokine-monopotent IL7Rα^+^ cells expressing lymphoid-directed CXCR3, CXCR4, CCR6, CCR7 and CCR9, but not the myeloid-directed chemokine receptors. In this model we propose that the monopotent CSF-1R^+^ progenitor pool also contains AGM-pHSC-derived myeloid progenitors, which should be unable to generate erythrocytes. The AGM-pHSC-derived progenitors change their location at E14.5 from LYVE-1^high^ endothelium by transmigration to ALCAM^high^ mesenchyme. After E13.5 IL7Rα^+^ CXCR3^+^CXCR4^+^CXCR5^+^ B-lineage progenitors expand exponentially, while AGM-pHSC-derived progenitors are found in unchanged numbers between E13.5 and 16.5.

The IL7Rα^+^CSF-1R^+^ cells also failed to produce erythrocytes (Fig. 3). Correspondingly, GATA-1 and PU.1 transcripts were only detected at high levels in the erythro-myeloid CSF1-R^+^ progenitors (Fig. 2). These differences in the differentiation capacities and transcription factor expression might suggest a difference in their hematopoietic origins. It is reasonable to expect from our and previous results^13^ that the IL7Rα^+^CSF^−^1R^+^ cells (10^4^ at E13.5), the IL7Rα^+^ cells (10^4^ at E13.5), and a minority of the CSF-1R^+^ cells (maybe as little as 10^4^ at E13.5) derive from the AGM-derived definitive pHSC-dependent hematopoiesis. By contrast, the majority of the CSF-1R^+^ subset (10^5^ at E13.5) develops as YS-derived erythro-myeloid progenitor lineage. A further dissection of these subsets regarding their gene expression such as specific globins, surface markers and transcription factors, and lineage tracing experiments^13^ are needed to clearly identify and verify the embryonic site of origin of these progenitor subsets, which we propose in our model (Fig. 7).

Our results suggest that the AGM-derived progenitors, which are reactive to two cytokines restrict their developmental capacities, as they reduce their cytokine receptor expression to IL7Rα^+^ or CSF-1R^+^. Therefore, chemotaxis might guide these progenitors to specific chemokine- and cytokine-producing tissue sites where this lineage restriction can be coordinated. Our results show for the first time that the lympho-myeloid IL7Rα^+^CSF1-R^+^ progenitors express two overlapping sets of chemokine receptors, which provide potential responsiveness to lymphoid- as well as myeloid differentiation. Once committed to either of the two lineages the lymphoid restricted IL7Rα^+^ progenitors retained only lymphoid-directed chemokine receptor expressions, while the erythro-myeloid CSF-1R^+^ progenitors only expressed an exclusive set of myeloid-directed chemokine receptors.

However, the responsiveness of most of these chemokine receptors was not detectable in *in vitro* transwell migration assays. Only CXCL12 stimulated a strong migration response of all three types of progenitors – all expressing CXCR4. Also CCL19 and CXCL11 induced considerable migration of all three progenitors *in vitro*, although with at least 10-fold reduced migration indices, compared to CXCL12. These results and our previous studies^41^ leave us to propose that CXCL10/11 and CCL19, produced by LYVE-1^high^ endothelial cells are active in the transendothelial migration of all three lymphoid-myeloid progenitors at E13.5 (Model in Fig. 7). Lyve-1 expression during mouse embryonic development is different from Lyve-1 expression characterizing lymphatic vessels in peripheral lymph nodes of the adult mouse. In fact, previous studies^45^,^46^ show that Lyve-1 is expressed throughout the embryonic blood vasculature (e.g. in arterial endothelial cells of fetal liver), more as a marker of immature vessels than of mature, peripheral lymphatic endothelium. Therefore, these studies support our model (Fig. 7) that the hematopoietic progenitors transmigrate from embryonic blood through Lyve-1+ endothelium to the mesenchymal environments inside.

Upon transendothelial migration the progenitors are chemoattracted within the fetal liver to the CXCL12-producing ALCAM^high^ mesenchymal liver environment, where SCF, IL7 and CSF-1 are produced to induce lineage differentiation. For further differentiation along the B-lineage pathway CXCL10 and CXCL11, produced by ALCAM^high^ cells, might cooperate with CXCL12, while CCL5 and CX3CL1 might do so in further myeloid differentiation in additional microenvironments. The response of the progenitors to the chemokines might change from gradient migration at E13.5-E14.5, to residence and cooperation in inductive processes of differentiation in specific cytokine-producing niches at later days of development. Our results suggest that different ALCAM^high^ mesenchymal environments might produce different chemokines and cytokines such as CXCL10/11 and IL7 for B-lymphoid, CCL5 and CSF-1 for myeloid differentiation. However, we have not yet been able to identify specific sub-compartments of these ALCAM^high^ mesenchymal cells.

CCL21 expression was not detectable between E13.5 and E16.5 in fetal liver, although both the IL7Rα^+^CSF-1R^+^ and the IL7Rα^+^ progenitors expressed the corresponding receptor, CCR7 and were responsive to CCL21. Since CCL21, CCL25 and CCL19 have previously been detected in fetal thymus^47^,^48^ we confirmed their expression by RT-PCR in fetal thymus developing from E13.5 to E16.5 (data not shown). Thymic EpCAM/ALCAM^+^ epithelial cells expressed CCL19, CCL21 and CCL25, as well as IL7. Therefore, it appears that lymphoid and lympho-myeloid progenitors have thymus-directed chemotactic capacities which are not used in fetal liver. It remains to be investigated whether these progenitors actually migrate from liver to thymus or from thymus to liver, to make use of their T- or B- lymphoid differentiating capacities in the appropriate environment.

Since several of the tested chemokines did not induce a significant response in the transmigration *in vitro* assays, it indicates that the expression of a chemokine receptor on the surface of a cell does not directly correlate with its ability to migrate towards this chemokine in a soluble form as it was shown for CXCL12, CXCL10/11, CCL21 and CCL19. Future analyses will have to elucidate possible functions of these chemokines in fetal hematopoiesis beyond the migration, such as cooperation, adhesion, residence, proliferation, differentiation and survival.

Taking into account our analyses of chemokine receptor expression and chemotaxis of lympho-myeloid progenitors, and of chemokine production in endothelium and mesenchyme, and previously published studies^11^,^13^ we propose a model of lympho-myeloid development in the murine fetal liver between E13.5 and E16.5, shown in Fig. 7. At E13.5, and until E16.5, the most frequent progenitors within the liver are erythroid-myeloid progenitors (EMP) which develop from E7.5 extraembryonically in the yolk sac. The IL7Rα^+^CSF-1R^+^ progenitors are 10-fold less frequent and derive presumably from the intraembryonic AGM region starting at E10.5. It remains an open question if these IL7R^+^CSF1-R^+^ cells give rise directly to monopotent IL7Rα^+^ and also contribute to the CSF1-R^+^ subsets.

Our earlier analyses^41^, which did not distinguish between bipotent and monopotent IL7Rα^+^ progenitors, detected at E13.5, 75% of IL7Rα^+^ progenitors near LYVE-1^high^ endothelium. At E14.5 >50% of B cell precursors (SLC^+^) were found next to ALCAM^high^ mesenchymal cells, where they entered IL7- and SCF-stimulated exponential proliferation observed until 16.5. Unfortunately, despite repeated efforts, we have not yet been able to detect IL7Rα^+^CSF-1R^+^ progenitors in immunohistochemical sections. But as we showed, the absolute numbers of all analysed progenitor subsets remain nearly constant between E13.5 and E16.5. It is, therefore, reasonable to expect that the stromal microenvironment of the fetal liver continuously initiates lineage commitment, providing the required chemokines and cytokines. We assume that the endothelial Lyve-1^high^ and the mesenchymal ALCAM^high^ cells remain competent to support homing of progenitors and their lineage differentiation from early to late days of fetal liver development, although *in vitro* migration capacities of progenitors decline with increasing time of embryonic development. In conclusion, it appears that fetal liver is competent between E13.5 and 16.5 to continuously initiate B-lymphopoiesis and myelopoiesis. This changes our earlier conclusions that B-lymphopoiesis occurs in one wave^49^, and now describes this development as a continuous process between E13.5 and E16.5 from cytokine-bipotent, chemokine polyreactive progenitors over IL7Rα^+^ monopotent, chemotactically restricted progenitors to pro and preB cells. During this differentiation towards B-lineage commitment in fetal liver the progenitors retain an expression program for chemokine receptors which, if challenged by the proper chemokine, produced e.g. in the thymus, would allow them to enter T-lymphocyte lineage commitment.

## Material and Methods

### Animals

Mice were bred and maintained under specific pathogen free conditions, and all experiments were performed according to institutional guidelines and German Federal laws on animal protection. C57BL/6J (Ly5.2) mice (8–10 weeks old) were obtained from Charles River, Wilmington, MA. Embryos were obtained by timed mating. The morning of the detection of vaginal plugs was considered as E0.5. Animal protocols were approved by Landesamt für Gesundheit und Soziales, Germany (T0079/13).

### Embryo dissection and cell preparations

Embryos were removed from decidua and Reichert’s membrane and dissected in phosphate buffered saline (PBS). Single cell suspensions were incubated with an anti-CD16/32 (FcγRII, 2.4G2) antibody to block unspecific binding, followed by specific antibody combination staining.

### Flow cytometry and cell sorting

Cells were stained for surface markers followed by flow cytometry analysis using a BD LSR II (BD Biosciences, San Diego, CA) and data analysis using FlowJo software (Tree Star, Ashland, OR).

Antibodies used were FITC-anti-CD3 (17A2), -CD19 (eBio1D3), -B220 (RA3-6B2), -F4/80 (BM8), -Gr1 (RB6-8C5), APC-anti-CD115 (AFS98), PE-anti-IL7Rα (eBioSB/199), PeCy5-Flt3 (A2F10). DAPI (Invitrogen; D1306) was used as cell viability dye.

Preparative cell sortings were performed on BD FACS Aria II or BD Influx. Purity check was performed after sorting and sorted fractions with ≥90% purity were used for further experiments.

### RT-PCR and real-time RT-PCR

Total RNA was isolated from sorted cells using Trizol (Invitrogen). RNA was reverse transcribed using the SuperScript Reverse Transcriptase III (Invitrogen, Carlsbad, CA). Quantitative real-time PCR was carried out by an ABI StepOnePlus thermal cycler (Applied Biosystems, Foster City, CA) using SYBR Green. Differences in cDNA input were compensated by normalizing against GAPDH expression levels and changes in gene expression were calculated using the ΔΔCt method. Primer sequences are provided in Supp. Table 1.

### *In vitro* culture

For B cell differentiation CSF-1 deficient OP9 stromal cells (a kind gift of Dr. Zúñiga-Pflücker, University of Toronto) were seeded in 96-well plates, grown to 70% confluency and irradiated with 30 Gy. Sorted progenitors were seeded in alphaMEM supplemented with 1% IL7 conditioned supernatant and 2%FCS. Granulocyte and F4/80^+^ macrophage differentiation was monitored under same culture conditions.

For osteoclast differentiation CSF-1 producing ST2 cells were seeded in 24-well plates, grown to 90% confluency. Sorted progenitors were seeded in alphaMEM with 10%FCS, dexamethasone and vitamine D. After 6 days cells were fixated with formalin followed by 50% ethanol/50% acetone and stained for TRAP-expression^50^.

For T cell differentiation DL1-OP9 stromal cells and 0.5% of IL7 and 2% of Flt3L conditioned supernatants were used^51^.

Cytokine supernatants were produced by using the appropriate hybridoma cell lines: IL-7 (J558L/IL-7)^52^, SCF (CHO-SCF, a kind gift of Dr. Thorsten Feyerabend, Universität Ulm)^53^, Flt-3L (Sp2.0-Flt3-L, a kind gift of Dr. Paulo Vieira, Institute Pasteur, Paris)^54^.

### Migration Assays

Chemotaxis assays were performed across bare polycarbonate membranes^55^. Briefly, a total of 100 μl containing 1 × 10^5^ sorted lin^neg^ (CD3, CD19, Gr1, F4/80, Ter119) fetal liver cells were transferred to the top chamber of 5 μm pore polycarbonate membrane (Corning, Acton, MA) while the lower chamber contained 100 ng/mL of the specific chemokine in media (RPMI1640 supplemented with 0.5% BSA). After 3 hours of incubation at 37 °C and 5% CO2, transmigrated cells were counted and analyzed by flow cytometry. All experiments were performed in triplicates. Data were normalized as the migration index: the number of migrating cells in an experimental chamber divided by the number of migrating cells in control chamber without addition of chemokines. FACS analysis of sorted fractions before and after 3 hours of incubation with migration media was performed to confirm that the cells did not change their phenotype within the experiment duration.

### Statistical analysis

Statistical analyses were performed on all numerical data as indicated in each figure using GraphPad Prism software (GraphPad, San Diego, CA). Unless otherwise stated, the statistical significance of differences was calculated depending on the experimental setup using a one-way or two-way ANOVA test with multiple comparisons (Turkey or Bonferroni correction). P values less than 0.05 were considered significant (*). All data are presented as mean ± SEM or mean ± SD.

## Additional Information

**How to cite this article**: Kajikhina, K. *et al.* Chemokine polyreactivity of IL7Rα^+^CSF-1R^+^ lympho-myeloid progenitors in the developing fetal liver. *Sci. Rep.* **5**, 12817; doi: 10.1038/srep12817 (2015).

## Supplementary Material

Supplementary Information
