# Supplementary material for: Chemokine polyreactivity of IL7Rα+CSF-1R+ lympho-myeloid progenitors in the developing fetal liver
Source: Sci Rep. 2015 Aug 3;5:12817. doi: 10.1038/srep12817 (PMC4522655; doi:10.1038/srep12817)
Supplement: Supplementary Information [file srep12817-s1.pdf]

Chemokine polyreactivity of IL7Rα$^+$CSF-1R$^+$ lympho-myeloid progenitors in the developing fetal liver

Katja Kajikhina[1], Fritz Melchers* [1], Motokazu Tsuneto[1,2]

1: Max Planck Institute for Infection Biology, Chariteplatz 1, 10117 Berlin, Germany

2: Current address: Reproductive Centre, Mio Fertility Clinic, 2-1-1 Kuzumo-minami, Yonago, Japan.

Supplementary information

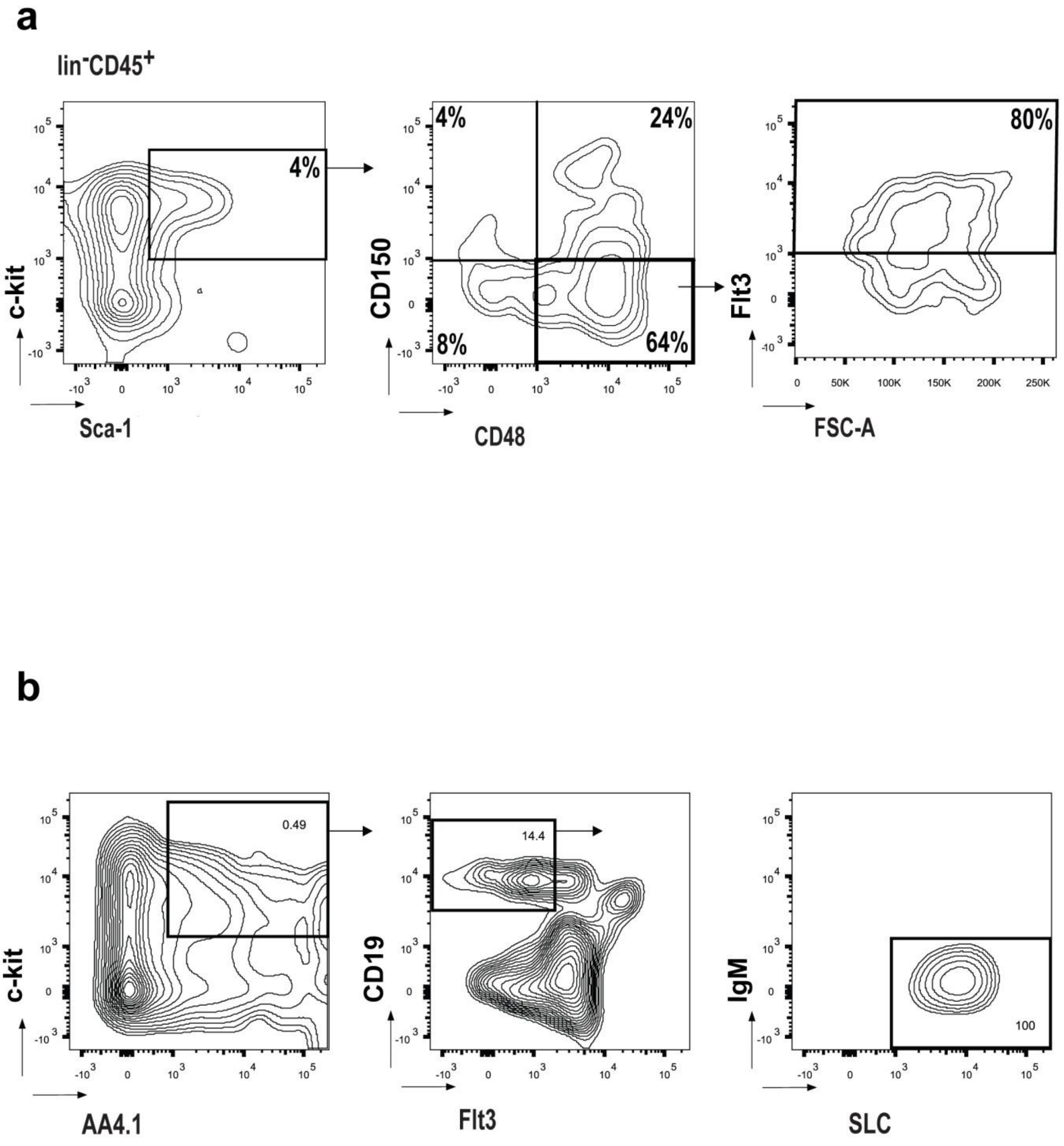

Supp. Fig. 1: Staining of pHSC and preB cell subsets in the developing murine fetal liver. (a) Gating strategy for the pHSC staining, staining shown from fetal liver E13.5 **(b)** Gating strategy for the preB cell staining, staining shown from fetal liver E15.5

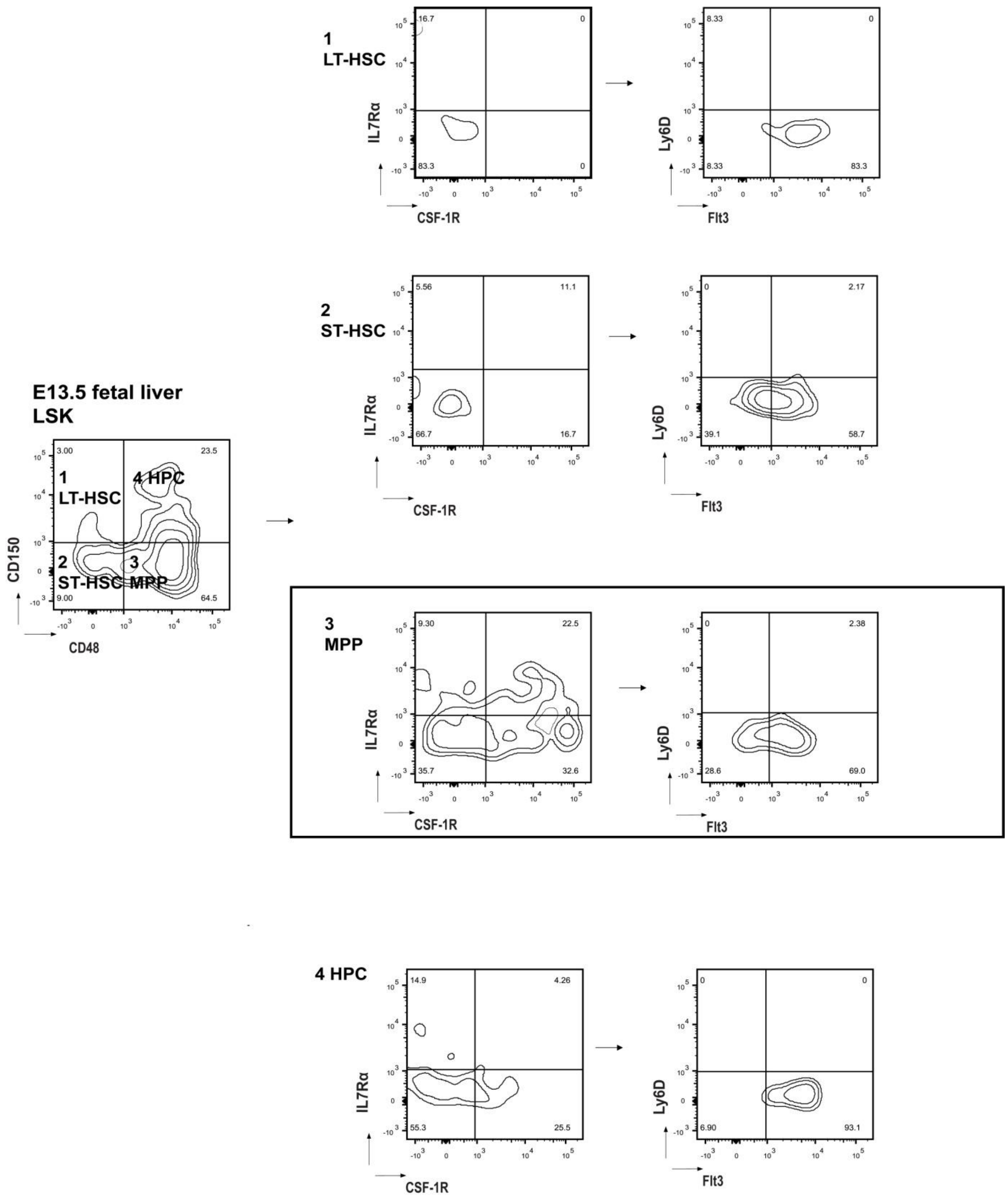

Supp. Fig. 2: Hematopoietic stem cell and progenitor staining using SLAM markers shows heterogeneity within the LSK CD150$^-$CD48$^+$ MPP subset regarding expression of IL7Rα and CSF1-R. Total fetal liver was stained at E13.5. One representative experiment is shown out of n≥6.

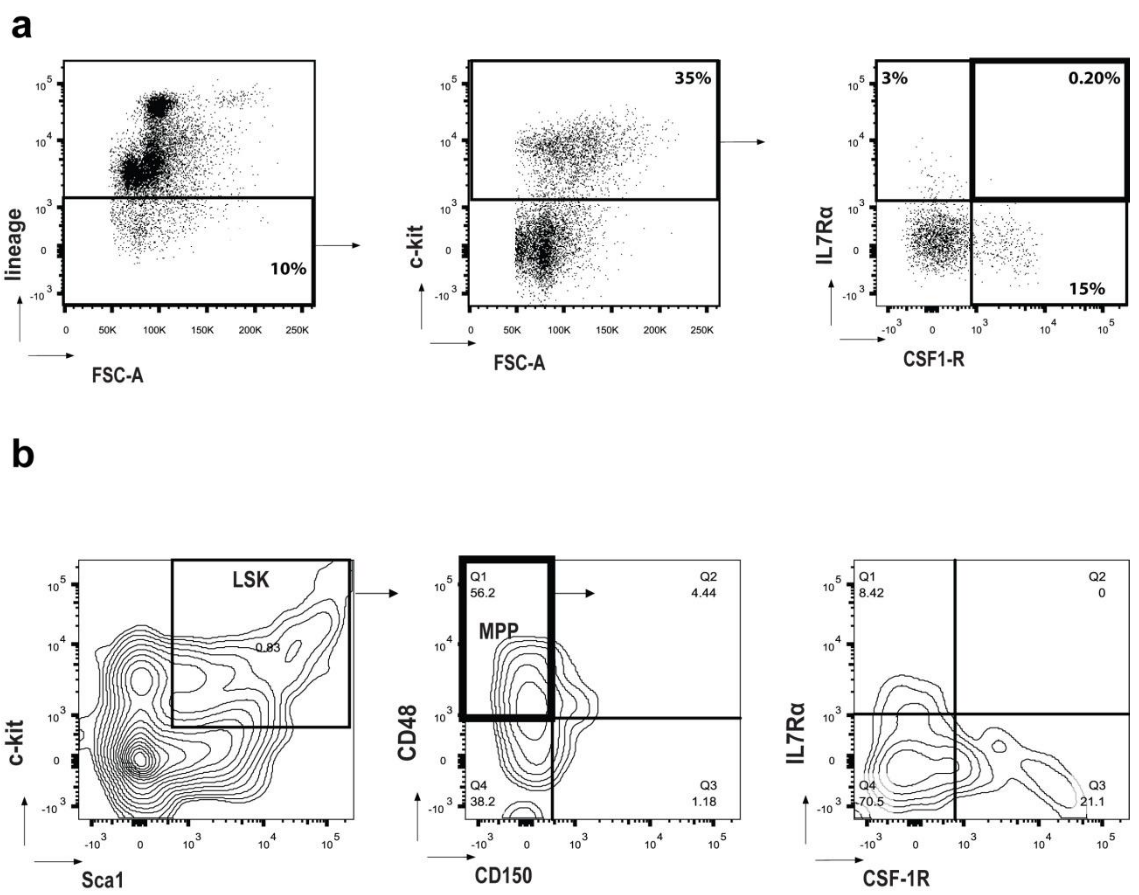

c

Subset	Tissue	Absolute number per $1x10^7$ cells	Percentage of total cells
fLMPP	E13.5 fetal liver	$2x10^4$	(0.25%)
	E16.5 fetal liver	$3x10^3$	(0.03%)
	Adult BM	$5x10^2$	(0.002%)
MPP	E13.5 fetal liver	$2x10^4$	(0.25%)
	E16.5 fetal liver	$2x10^3$	(0.025%)
	Adult BM	$5x10^4$	(0.45%)
fLMPP $Sca1^{neg}$	E13.5 fetal liver	$2x10^3$	(0.02%)
	E16.5 fetal liver	$5x10^1$	(0.005%)
	Adult BM	0	0

Supp. Fig.3: Comparison between the "classic" MPP compartment and the analysed fLMPP subset in the adult bone marrow (a) FACS profile of the fLMPP subset as analysed in the fetal liver shows that only very few cells of this phenotype are detectable in adult bone marrow; (b) FACS profile of the "classic" MPP compartment in adult bone marrow; (c) calculation of absolute and relative frequencies in adult bone marrow compared to the fetal liver of both fLMPP and MPP subsets as well as of the fLMPP-$Sca1^{neg}$ subpopulation that is missing within the MPP subset.

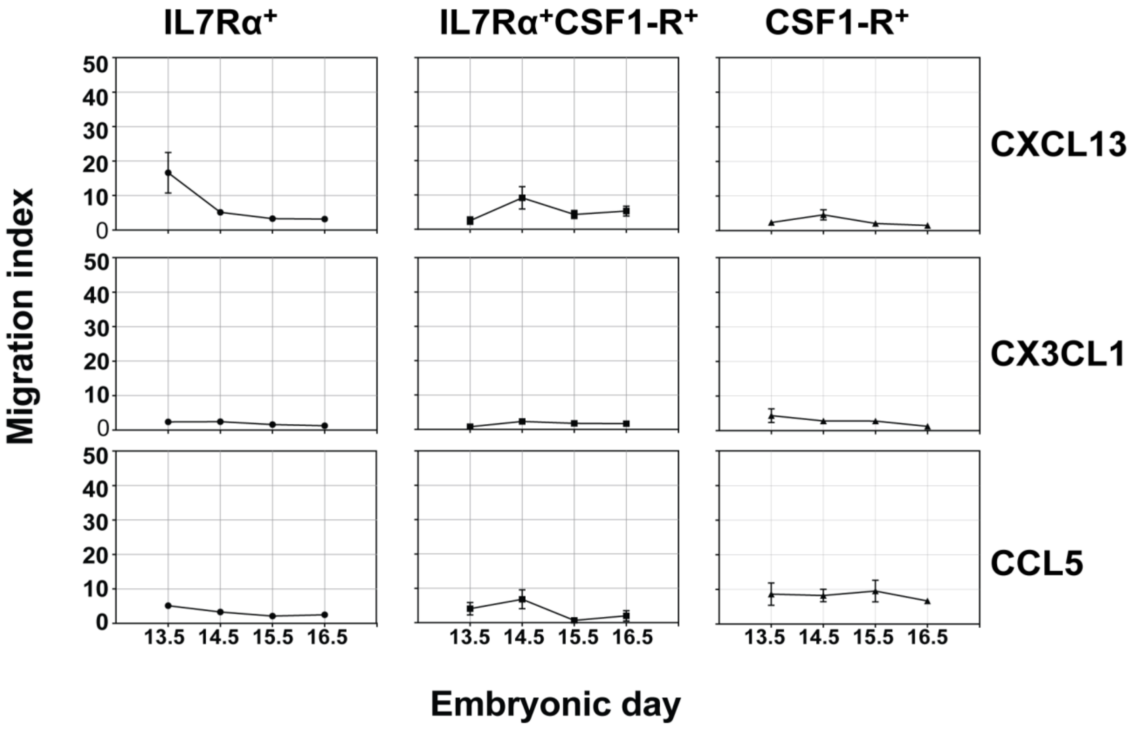

Supp. Fig.4: Chemoattraction assays show no significant responses to recombinant chemokines CXCL13, CX3CL1 and CCL5 in early progenitors between E13.5 and E16.5. Total 10^5 lin^{neg} cells from fetal livers between E13.5 and E16.5 were inserted into a transwell chamber to assay the *ex vivo* migration capacity of early progenitors to recombinant chemokines (100ng/ml). The number of migrated cells was normalized to the number of migrated cells in absence of any chemokine in the same experiment (migration index). A two-way ANOVA test with multiple comparisons was performed to test the statistical significance (CI 95%).
